# Turning Up the Heat: Inflammasome Activation by Fungal Pathogens

**DOI:** 10.1371/journal.ppat.1004948

**Published:** 2015-07-23

**Authors:** Aldo Henrique Tavares, Pedro Henrique Bürgel, Anamélia Lorenzetti Bocca

**Affiliations:** Laboratório de Imunologia Aplicada, Departamento de Biologia Celular, Instituto de Biologia, Universidade de Brasília, Brasília, Distrito Federal, Brasil; Duke University Medical Center, UNITED STATES

## The Inflammasome

Since its first description in 2002 [1], the inflammasome has been implicated in the mechanisms underlying a growing number of infectious, autoimmune, and metabolic diseases [2]. Regarding infectious processes, several studies have shown the involvement of this critical component of innate immunity in the outcome of infection with nearly every class of microbe, including fungi [3]. Innate immunity is the frontline of defense against infection and relies on the ability of its main players (phagocytes and epithelial barriers) to detect conserved components of microbes or pathogen-associated molecular patterns (PAMPs). In fungi, the carbohydrate polymers of the cell wall, such as chitin, β-glucan, and mannan are the major PAMPs recognized by the host’s innate immune cells; this recognition occurs via germline-encoded receptors termed pattern recognition receptors (PRRs) [4]. In addition to PAMPs, endogenous molecules associated with damaged host cells, or damage-associated molecular patterns (DAMPs), are released during tissue injury and activate PRRs. This innate detection system includes the Toll-like receptors (TLRs), C-type lectin receptors (CLRs), RIG-I-like receptors (RLRs), NOD-like receptors (NLRs), and AIM2-like receptors (ALRs). Although the main fungal-recognition PRRs (CLRs and TLRs) are bound to the cytoplasmic membrane of innate immune cells [4], fungal sensing by PRRs located in the cytosol, such as the NLRs and ALRs, is becoming increasingly evident.

A number of NLRs and ALRs can assemble into the inflammasome, a multiprotein complex consisted of PRRs such as NLRP3 (NLR family, pyrin domain-containing 3), NLRC4, or AIM2, adaptor protein ASC (apoptosis-associated speck-like protein containing a caspase activation and recruitment domain (CARD), and procaspase-1 [3]. Upon formation of the complex, procaspase-1 is cleaved into an active cysteine protease, which further cleaves the proinflammatory cytokines IL-1β and IL-18 into their mature forms, followed by unconventional secretion. IL-1β and IL-18 mediate several innate antimicrobial responses and are critical to direct adaptive Th17/Th1 cellular responses [5]. In addition, inflammasome activation causes pyroptosis, a lytic inflammatory form of cell death [2,5].

## NLRP3 Inflammasome Priming by Fungal Pathogens

**Fig 1:**
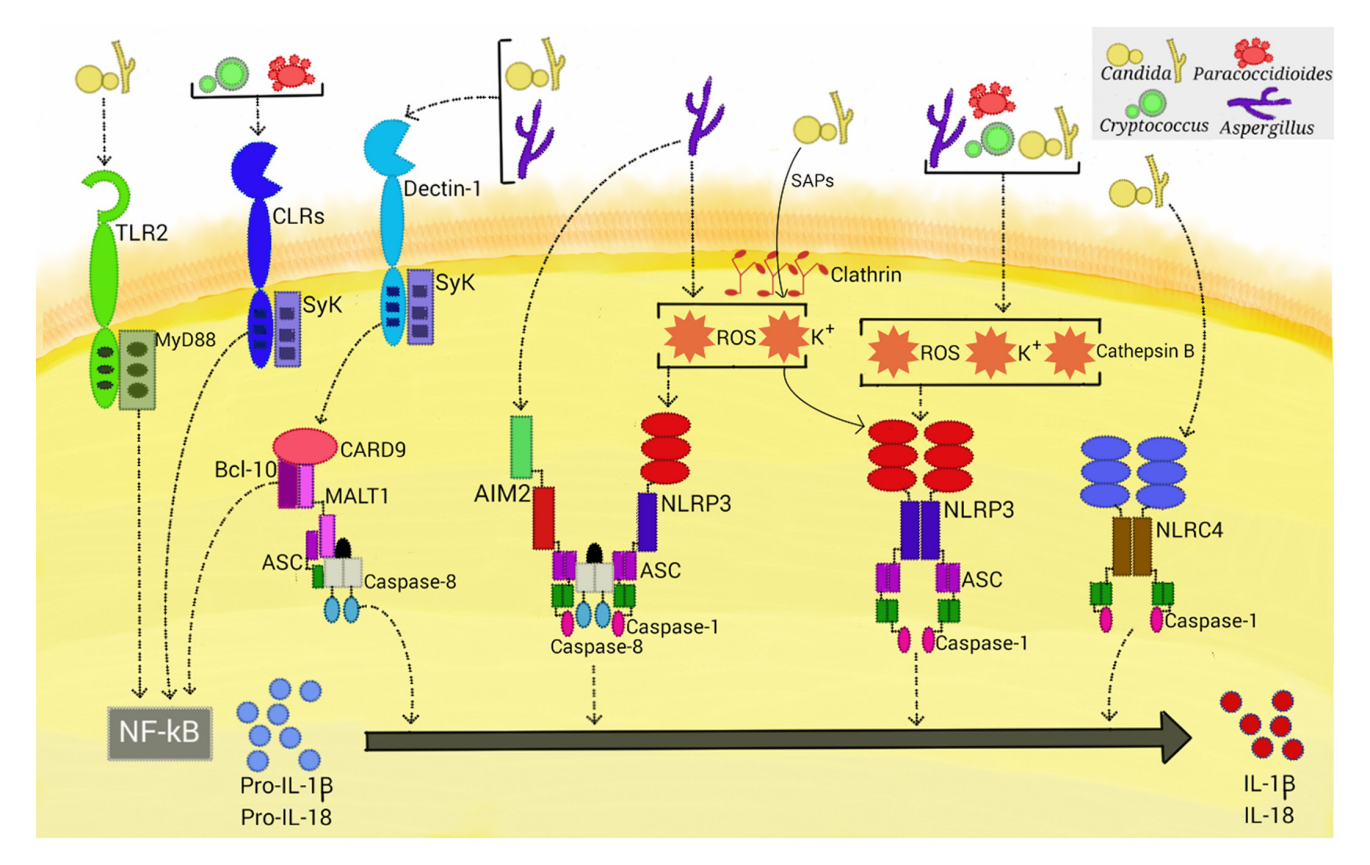
Activation of inflammasomes by fungal pathogens. Four inflammasome complexes are activated when engaged by systemic fungal pathogens. *Candida*, *Aspergillus*, *Cryptococcus*, and *Paracoccidioides* trigger priming-associated receptors (e.g., dectin-1 and TLR2), thereby promoting the first step (priming) of inflammasome activation. All four pathogens activate the canonical caspase-1-dependent NLRP3 inflammasome by inducing ROS, K^+^ efflux, and cathepsin B release. *Candida*-secreted aspartic proteases (Saps) are internalized and also activate the NLRP3 inflammasome via induction of K+ efflux and ROS production. *Aspergillus* activates both caspase-1- and caspase-8-dependent inflammasomes, requiring the simultaneous activation of the NLRP3 and AIM2 receptors. In terms of NLRP3-independent inflammasomes, *Candida* and *Aspergillus* activate a caspase-8-dependent inflammasome that requires the engagement of dectin-1, Syk signaling, and ASC recruitment. In addition, *Candida* triggers the NLRC4 inflammasome.

Among the inflammasomes, the NLRP3 inflammasome is the main one associated with fungal infection. In contrast to its counterparts, which only respond to a few specific PAMPs, the NLRP3 inflammasome is activated by a diverse array of unrelated triggers that include PAMPs from every class of pathogen, environmental irritants, and DAMPs. Although the precise mechanism of NLRP3 inflammasome activation is unclear, there is evidence suggesting that it is a two-step process [6]. The first, or priming, step is an NF-κB-dependent pathway that triggers expression of pro-IL-1β, pro-IL-18, and optimal NLRP3. In the second, or activation, step, assembly of the inflammasome complex leads to caspase-1 activation to promote cleavage of the immature cytokines. Priming is most frequently achieved via PRR recognition of PAMPs. In this manner, fungal PAMPs are recognized by several CLRs and TLRs that can potentially activate NF-κB [4]. However, dectin-1-dependent signaling is emerging as the key pathway involved in fungus-induced NLRP3 priming (Fig 1) [7]. In addition, this PRR is necessary to activate a caspase-8-dependent inflammasome (see below). Dectin-1, the major β-glucan receptor, uses an immunoreceptor tyrosine-based activation motif to couple itself to Syk kinase for downstream signaling to NF-κB via CARD9-Bcl10-MALT1 (CBM) scaffold, resulting in cytokine production. In addition, phagocytosis and reactive oxygen species (ROS) production result from dectin-1 engagement [8]. The dectin-1 receptor is required for the production of pro-IL-1β in murine and human myeloid cells infected with *Candida albicans*, *Microsporum canis*, and *Malassezia* spp. [9–11]. Consistent with these results, mice deficient in dectin-1 and orally infected with *C*. *albicans* presented significantly reduced serum IL-1β levels [12]. Notably, mice lacking dectin-1 or CARD9 and humans with mutations in these proteins are susceptible to candidiasis [12–14]. Although the direct role of dectin-1 has not been evaluated, Syk-dependent NLRP3 priming also occurs in *Paracoccidioides brasiliensis*-, acapsular *Cryptococcus neoformans*- and *Aspergillus fumigatus*-infected cells [15–17]. Interestingly, glucuronoxylomannan, the main capsule component of *C*. *neoformans*, inhibits Syk signaling and ultimately NLRP3 inflammasome activation, which may facilitate the intracellular parasitism of this fungus [16]. Considering that the CLRs dectin-2 and mincle share the same downstream pathway as dectin-1, further studies will be necessary to assess the role of these receptors in NLRP3 priming by fungal pathogens.

In addition to the dectin-1/Syk pathway, TLR2 and the critical TLR signal adaptor protein MyD88 (Fig 1) are also necessary for the production of pro-IL-1β in murine macrophages infected with *C*. *albicans*. In contrast, priming of murine dendritic cells stimulated with this fungus only required the dectin-1/Syk pathway [9,18]. These findings are consistent with those of earlier studies that demonstrate that cooperative signaling between dectin-1 and TLR-2 is necessary for TNF-α production by macrophages stimulated with zymosan (dectin-1 and TLR2 agonist), whereas in dendritic cells, dectin-1 signals directly induce cytokine production [19]. Thus, the simultaneous engagement of CLR and TLR is differentially regulated in macrophages and dendritic cells, suggesting a mechanism by which myeloid cells can be fine-tuned to regulate the inflammatory process.

## NLRP3 Inflammasome Activation by Fungal Pathogens

The mechanisms by which the NLRP3 inflammasome senses its vast array of activators seem to converge on several cellular disturbances that are probably nonexclusive, such as potassium (K+) efflux, calcium influx, ROS production, and the occurrence of cytosolic cathepsins derived from lysosomal disruption [6]. Although there are several controversies surrounding the actual role of each factor in inducing NLRP3 inflammasome assembly, it has been shown that fungi generally induce ROS, K+ efflux, and lysosomal rupture-dependent IL-1β release (Fig 1). ROS, which are a conserved danger signal, and K+ efflux are required by nearly all fungal species found to activate the NLRP3 inflammasome to date. Interestingly, dectin-1/Syk-mediated release of ROS was demonstrated to be critical in inflammasome activation in *C*. *albicans*-infected murine and human phagocytes [18]. Thus, Syk-coupled dectin-1 signaling is implicated in both priming and activation of the NLRP3 inflammasome. Recently, secreted aspartic protease (Sap) 2 and Sap6 from *Candida* were the first fungal proteins demonstrated to provide a caspase-1-dependent NLRP3 inflammasome activation signal (Fig 1) [20]. The inflammasome activation required Sap internalization via a clathrin-dependent mechanism, followed by induction of K+ efflux and ROS production. Additionally, these proteases activate, through the induction of type I IFN production, caspase-11 that cooperates with caspase-1 to maximize IL-1β maturation [21]. In view of the elevated secretion of Saps during vaginal candidiasis, studies are necessary to assess the in vivo relevance of these data.

Although internalization and cytoplasmic leakage of cathepsin B due to lysosomal disruption are common features of particulate activations of the NLRP3 inflammasome, data on the role of cathepsin B in fungal inflammasome activation are conflicting. Pietrella et al. [20] and Joly et al. [22] found this intracellular perturbation to be necessary in macrophages infected with *C*. *albicans*, whereas no role was found in dendritic cells. In human monocytes infected with *C*. *neoformans*, *M*. *canis*, and *Trichophyton schoenleinii*, cathepsin B release is required for IL-1β maturation [13,23,24]. In this manner, differential requirements for inflammasome activation, as shown for PRR usage in the priming step, may exist in these cells.

Morphogenesis is another important aspect of inflammasome activation by fungal pathogens. It is well established that only *A*. *fumigatus* hyphal fragments, and not conidia, activate the NLRP3 inflammasome [17]. A more complex story emerges from *Candida* studies. Hyphal formation was initially thought to be essential for the activation of the NLRP3 inflammasome [22]. However, studies showing *Candida* mutants that lack the ability to form hyphae but are able to trigger the inflammasome strongly suggest that filamentation per se is not the only responsible for inflammasome activation [25]. In fact, exposition of PAMPs, such as β-glucan and chitin, is the critical factor for NLRP3 inflammasome activation, even though this process is likely to be coordinately regulated with filamentation [26]. These data support recent studies showing that remodeling of the *Candida* cell wall, but not hyphal formation, upon internalization by host macrophages is essential for caspase-1/NLRP3-mediated pyroptosis [27,28].

## Beyond the NLRP3 Inflammasome in Fungal Infections

In addition to the NLRP3 inflammasome, a NLR-independent caspase-8-dependent inflammasome and other canonical caspase-1-dependent inflammasomes are activated when stimulated with fungi (Fig 1). Gringhuis et al. [29] found that human dendritic cells stimulated with certain strains of *C*. *albicans* did not utilize caspase-1 to process IL-1β. This finding led to several experiments demonstrating a NLR-independent dectin-1/Syk-dependent inflammasome activation route, with the assembly of the CBM scaffold and processing of IL-1β mediated by recruitment of MALT-1/caspase-8 and ASC into this complex. This route was also activated by other species of *Candida* and different *A*. *fumigatus* strains. Moreover, the dectin-1-mediated activity, in contrast to NLRP3 inflammasome priming, did not require phagocytosis, suggesting a direct extracellular sensing mechanism. Later, Ganesan et al. [30] demonstrated that caspase-8, dectin-1, and CR3, another receptor implicated in β-glucan sensing, are necessary for IL-1β processing by murine dendritic cells infected with *C*. *albicans*. Interestingly, caspase-8 activation in these studies raises questions about *Candida*-induced programmed cell death pathways, as caspase-8 also has a significant role in initiating apoptosis [31].

Best known for its role in inflammatory responses to flagellated and type 3 secretion system-expressing bacteria, the NLRC4 inflammasome had been found to be dispensable for canonical IL-1β maturation in macrophages and dendritic cells challenged with *C*. *albicans* [18]. In contrast, Tomalka et al. [32] showed activation of the NLRC4 inflammasome, along with NLRP3, in a murine model of oral *C*. *albicans* infection. One plausible explanation for this discrepancy is that *Candida*-induced alterations in the mucosal barrier may have allowed bacterial pathobionts to penetrate and infect cells and activate the NLRC4-dependent inflammasome. Interestingly, *A*. *fumigatus* induces cooperative and synergistic activation of the NLRP3 and AIM2 inflammasomes in dendritic cells and in a mouse intranasal infection model [33]. Upon ligand binding, these inflammasome receptors trigger the assembly of a single platform, containing ASC, caspase-1, and caspase-8, which are required for IL-1β and IL-18 processing. This study also corroborated the cytoplasmic disturbances (i.e., ROS and K+ efflux) induced by *A*. *fumigatus* that are necessary for NLRP3 activation [17] and suggested the availability of fungal dsDNA (the main AIM2 ligand) in the cytosol of infected cells. Considering that bacterial RNA and DNA are sensed by NLRP3 [34], it is possible that NLRP3 and AIM2 both recognize a common nucleic acid composition of *A*. *fumigatus*, prompting the formation of a cooperative inflammasome. Altogether, these studies highlight the emerging trend that multiple inflammasome receptors can be activated upon infection with fungal pathogens, as has been shown for several species of bacteria, adding further complexity to the fungus–host relationship.

## The Inflammasome and Host Resistance against Fungal Pathogens

The depletion of inflammasome components, inflammasome products, and relevant first-signal receptors has been linked to susceptibility of the host to several bacterial infections. Although the available data are scarce, it seems that this phenomenon extends to fungal infections. In *C*. *albicans*, it has been demonstrated that in a disseminated model of infection, loss of NLRP3 leads to increased mortality and an increased fungal burden in several organs, such as the kidney, lung, and liver [18,22]. van de Veerdonk et al. [35] further showed an essential role for ASC and caspase-1 in regulating adaptive antifungal immune responses and host survival during disseminated candidiasis through the induction of Th1 and Th17 development. NLRP3 inflammasome components were also studied in a model of mucosal *Candida* infection, with NLRP3-, ASC-, or caspase-1-deficient mice being more susceptible to invasive disease, given that they presented a higher fungal burden in the kidneys and the digestive system [9]. Furthermore, loss of the IL-1 receptor (IL-1R) and the priming-associated receptors dectin-1 and TLR-2 aggravated the infection. Using bone marrow chimeras, it was demonstrated that NLRP3 and NLRC4 have a protective but tissue-specific role in oropharyngeal candidiasis [32]. In contrast to NLRP3, whose activation is protective in both the hematopoietic and the stromal compartments, NLRC4 is essential in only the stromal compartment, where its activity is necessary for the induction of neutrophil influx into infected tissues and the avoidance of fungal dissemination, particularly early in infection. Nevertheless, these results should be interpreted with caution since it is also possible that mice lacking NLRC4 possess a diverse microbiota from other controls. In fact, microbiota differences are observed in inflammasome knockout mice [36], resulting in microbiota-driven differences in phenotypes (i.e., microbiota would track with the stromal compartment in bone marrow transplants). Recently, using an invasive pulmonary aspergillosis model, Karki et al. [33] showed that redundant activation of the NLRP3/AIM2 platform is essential for host protection, as it significantly limits dissemination of *A*. *fumigatus* hyphae from inflammatory foci. In addition, AIM2 and NLRP3 activity in both the hematopoietic and the stromal compartments is required to protect the host against aspergillosis. It was also shown that loss of key inflammasome-related cytokines (e.g., IL-1β and IL-18) is detrimental for the host. Regarding cryptococcosis, regardless of the route of infection (e.g., intraperitoneal or intranasal), mice lacking NLRP3 or ASC presented poorer survival compared with wildtype mice [23]. In fact, even for intranasal infection with a low virulence acapsular strain of *C*. *neoformans*, NLRP3 was required for effective lung leukocyte infiltration and fungal clearance. Finally, our group demonstrated that the presence of the IL-1R-dependent signaling, and NLRP3 is required to control the intracellular growth of *P*. *brasiliensis* within macrophages [15].

These studies suggest that the activation of inflammasomes is of vital importance for innate immunity to constrain the growth and dissemination of fungal pathogens, conferring fungal resistance upon the host. In support of this notion, patients diagnosed with vulvar vestibulitis syndrome present a length polymorphism in intron 4 of the NLRP3-coding gene, which has been associated with recurrent vulvovaginal candidiasis [37]. It is important, however, to keep in mind that fine-tuning of inflammasome activity is necessary to avoid intense and detrimental inflammatory responses. In fact, in a study utilizing a pulmonary aspergillosis model, Moretti et al. [38] suggested that the activation of the NLRP3 inflammasome may causes pathological sequelae in the face of an unresolved infection.

In summary, significant progress has been made in understanding the role of inflammasomes in fungal diseases. The activation of noncanonical inflammasome and multiple inflammasomes as well as the induction of pyroptosis being the most recent. However, questions remain unanswered. For example, what is the exact mechanism by which NLRP3 is activated and regulated by diverse fungal pathogens? What is the functional relevance of pyroptosis? Also poorly understood are the fungal evasion strategies developed to avoid inflammasome recognition and the inflammasome functionality in nonmyeloid cells during fungal infections. Clearly, it will be exciting to witness the future studies on those topics.
